# Heart failure after treatment for breast cancer

**DOI:** 10.1002/ejhf.1620

**Published:** 2019-11-12

**Authors:** Naomi B. Boekel, Fran K. Duane, Judy N. Jacobse, Michael Hauptmann, Michael Schaapveld, Gabe S. Sonke, Jourik A. Gietema, Maartje J. Hooning, Caroline M. Seynaeve, Angela H.E.M. Maas, Sarah C. Darby, Berthe M.P. Aleman, Carolyn W. Taylor, Flora E. van Leeuwen

**Affiliations:** ^1^ Epidemiology Netherlands Cancer Institute Amsterdam The Netherlands; ^2^ Medical Research Council Population Health Research Unit University of Oxford Oxford UK; ^3^ Nuffield Department of Population Health University of Oxford Oxford UK; ^4^ Medical Oncology Netherlands Cancer Institute Amsterdam The Netherlands; ^5^ Medical Oncology University Medical Center Groningen Groningen The Netherlands; ^6^ Medical Oncology Erasmus MC ‐ Cancer Institute Rotterdam The Netherlands; ^7^ Department of Cardiology Radboud University Medical Centre Nijmegen The Netherlands; ^8^ Radiation Oncology Netherlands Cancer Institute Amsterdam The Netherlands

**Keywords:** Breast cancer, Heart failure, Radiation dose–response, Anthracycline, Trastuzumab

## Abstract

**Background:**

We aimed to develop dose–response relationships for heart failure (HF) following radiation and anthracyclines in breast cancer treatment, and to assess HF associations with trastuzumab and endocrine therapies.

**Methods and results:**

A case–control study was performed within a cohort of breast cancer survivors treated during 1980–2009. Cases (*n* = 102) had HF as first cardiovascular diagnosis and were matched 1:3 on age and date of diagnosis. Individual cardiac radiation doses were estimated, and anthracycline doses and use of trastuzumab and endocrine therapy were abstracted from oncology notes. For HF cases who received radiotherapy, the estimated median mean heart dose (MHD) was 6.8 Gy [interquartile range (IQR) 0.9–13.7]. MHD was not associated with HF risk overall [excess rate ratio (ERR) = 1%/Gy, 95% confidence interval (CI) −2 to 10]. In patients treated with anthracyclines, exposure of ≥20% of the heart to ≥20 Gy was associated with a rate ratio of 5.7 (95% CI 1.7–21.7) compared to <10% exposed to ≥20 Gy. For cases who received radiotherapy, median cumulative anthracycline dose was 247 mg/m^2^ (IQR 240–319). A dose‐dependent increase was observed after anthracycline without trastuzumab (ERR = 1.5% per mg/m^2^, 95% CI 0.5–4.1). After anthracycline and trastuzumab, the rate ratio was 34.9 (95% CI 11.1–110.1) compared to no chemotherapy.

**Conclusions:**

In absence of anthracyclines, breast cancer radiotherapy was not associated with increased HF risk. Strongly elevated HF risks were observed after treatment with anthracyclines and also after treatment with trastuzumab. The benefits of these systemic treatments usually exceed the risks of HF, but our results emphasize the need to support ongoing efforts to evaluate preventative strategies.

## Introduction

Radiotherapy, anthracyclines and trastuzumab are commonly used treatments for breast cancer (BC) and have all been associated with an increased heart failure (HF) risk.^1^, ^2^, ^3^, ^4^, ^5^, ^6^, ^7^, ^8^, ^9^, ^10^ For radiotherapy there is evidence that ischaemic heart disease risk increases linearly with increasing mean heart dose (MHD).^11^ Evidence that it can increase HF risk is, however, conflicting.^1^, ^2^, ^3^, ^4^, ^10^, ^12^, ^13^, ^14^, ^15^ This may be due to differences in HF evaluation between the different studies, e.g. inclusion of only first cardiovascular disease (CVD) diagnoses vs. also those occurring after diagnoses of other CVDs, differences in the populations studied, the radiotherapy regimens used, or other cardiotoxic treatments given. Radiotherapy is known to cause impaired myocardial function in Hodgkin lymphoma survivors.^16^, ^17^ However, MHD is usually higher for Hodgkin lymphoma than that for BC. In a systematic review of MHD from BC radiotherapy during 2003–2013, average MHD was 5.4 Gy (range <0.1–28.6) in left‐sided and 3.3 Gy (range 0.4–21.6) in right‐sided BC.^18^ It remains unclear if this level of radiation exposure increases HF risk.

For anthracyclines there is strong evidence that HF risk increases with increasing cumulative dose.^19^ It usually occurs some years after treatment.^2^, ^13^, ^20^, ^21^, ^22^, ^23^ Trastuzumab also increases HF risk but, unlike anthracycline‐related HF, it usually occurs whilst patients are still receiving trastuzumab, and it mostly resolves once treatment is stopped.^7^, ^8^, ^9^

In this study we evaluated associations between radiation MHD and HF, and between anthracycline dose and HF. We also assessed HF associations with trastuzumab and for endocrine therapy.

## Methods

### Cohort population

A nested case–control study was performed within a Dutch cohort of long‐term BC survivors treated during 1970–2009. The cohort was identified through hospital‐based registries at the Netherlands Cancer Institute, Amsterdam and the Erasmus MC Cancer Institute, Rotterdam. Details of the data collection procedures for the patient cohort have been published previously,^2^, ^4^ and are described in online supplementary *Methods* *S1*.

### Cases and controls

Eligibility criteria for cases and controls were: (i) a diagnosis of BC stage I–IIIa between 1976 and 2009, (ii) age at BC diagnosis ≤65 years, (iii) no distant metastasis and no diagnosis of CVD that could have caused HF before cut‐off date (defined as date of HF diagnosis for cases, and for controls as the date of BC plus the time interval between BC and HF diagnosis for the corresponding case), (iv) no BC recurrence treated with radiotherapy, and (v) no thoracic radiotherapy for second malignancies other than primary BC before cut‐off date. (Thoracic) radiotherapy for recurrences or second malignancies was an exclusion criterion because MHD was not available for these regimens. Chemotherapy and/or endocrine therapy for a second malignancy or recurrence were allowed and taken into account in the total cumulative dose of specific drugs.

To include all cases with left ventricular systolic dysfunction, HF was defined as either dilated cardiomyopathy or HF with reduced ejection fraction (HFrEF) with ejection fraction (EF) <50%^24^ or a >10% drop from baseline EF (grade 2 according to adaption of the National Cancer Institute Common Terminology Criteria for Adverse Events; online supplementary *Methods* *S2*). In an attempt to exclude reversible HF, patients known to have a recovered EF ≤1 year after the first decrease of EF were excluded. CVDs were ascertained from medical charts and through questionnaires sent to the general practitioner of each patient in the cohort. For patients with clinical signs of HF, cardiologists were sent a questionnaire to confirm or reject the diagnosis. For each HF case, three controls were selected from the cohort, matched on age (≤5 years) and date of BC diagnosis (≤5 years). Individuals who had any CVD diagnosed at grade ≥2 before the cut‐off date were not included as controls.

Eligibility of each individual case was discussed (J.N.J., M.S., G.S.S., F.E.v.L.) taking into account all cardiovascular measurements and information, and blinded as to BC treatment. In total, 342 HF cases were identified, 102 were included, while 240 were excluded based on the eligibility criteria (online supplementary *Table* *S1*). Cases were permitted to serve as controls up to the date of HF development, and patients were allowed to serve as controls for multiple cases. In total, 306 controls were matched to cases, involving 290 unique individuals. Three patients were selected as both case and control, 11 controls served for two cases, and one control served for three cases.

### Data collection

Detailed treatment information was collected from oncology notes, including drug names, cumulative anthracycline dose, trastuzumab administration and type of endocrine therapy. Medical history at BC included: cardiovascular risk factors (diabetes, hypertension, smoking), and body mass index (BMI). Radiation charts were photocopied.

### Radiotherapy dosimetry

A ‘typical CT‐scan’ was used to estimate cardiac doses retrospectively for each woman in the study (online supplementary *Methods* *S3* and *Table* *S2*). In total, 45 regimens were identified and reconstructed on the ‘typical CT‐scan’. Dose distributions were generated for cobalt, electron and megavoltage beams using a three‐dimensional CT treatment planning system (Varian Eclipse™ version 10.0.39). Dose distributions from orthovoltage fields were generated using manual planning. The MHD, mean left ventricle dose, and the percent volume of heart receiving ≥5, ≥10, ≥20, and ≥25 Gy (V_5_ to V_25_) were estimated for each woman in the study using the total dose received as recorded from each individual radiotherapy chart and the dose–volume histogram of the regimen received.

### Statistical analyses

Chemotherapy was categorized into mutually exclusive treatment groups: no chemotherapy, cyclophosphamide, methotrexate, and 5‐fluorouracil‐like regimens, anthracycline‐based chemotherapy, anthracyclines plus subsequent trastuzumab, and other type of chemotherapy/unknown type. Cumulative isotoxic doxorubicin dose (mg/m^2^) for patients receiving epirubicin was estimated using a conversion factor of 0.55, i.e. 50 mg/m^2^ doxorubicin was considered equivalent to 90 mg/m^2^ epirubicin. Four mutually exclusive treatment categories were considered for endocrine therapy: no endocrine therapy, tamoxifen, tamoxifen and aromatase inhibitor, and aromatase inhibitor only. Endocrine therapy was only considered if it started ≥3 months before HF diagnosis/cut‐off date.

Rate ratios (RRs) for HF were estimated using logistic regression conditional on the matching variables, i.e. age at BC diagnosis (30–39, 40–49, 50–59, 60–65 years), year of BC diagnosis (1976–1979, 1980–1989, 1990–1999, 2000–2009), and follow‐up duration (<10, 10–19, 20–30 years). Confidence intervals (CIs) for categorical exposure variables were estimated for each category, including the reference category, from the amount of information in that category.^25^ HF is known to have different time dependence, aetiology and prognosis depending on the type of exposure. Therefore, in addition to analyses considering all the data, HF risk was assessed for each of the exposure radiotherapy, anthracycline and trastuzumab within separate categories of the other two exposures. For example, HF RRs were compared for groups of women with different anthracycline doses, separately for women who received radiotherapy but not trastuzumab, and for women who did not receive either radiotherapy or trastuzumab. For radiation and anthracyclines, dose–response relationships were estimated by modelling HF rate as K_m_(1 + βd) where d is MHD/doxorubicin equivalent anthracycline dose for individual patients, K_m_ is a constant specific to each matched set, and β is excess rate ratio (ERR, i.e. the proportional increase in HF rate) per unit increase in dose. Modelled cumulative HF incidences were estimated from the RRs together with the cumulative HF risk for the entire cohort. Analyses were performed using Stata (version 13.0, StataCorp LP, College Station, TX, USA) and Epicure (version 1.8, Hirosoft International) statistical software.

## Results

### Study population

In 102 cases of HF, the median age at BC diagnosis was 51 years (*Table* 1). Radiotherapy was received by 88/102 (86%) cases and 268/306 (88%) controls. Chemotherapy without trastuzumab was received by 43% cases and by 36% controls. Trastuzumab was received by 14% cases and 3% controls, nearly all of whom (21/24) also received anthracycline‐based chemotherapy. Patient characteristics by treatment are provided in online supplementary *Table* *S3*. Median age at HF diagnosis was 62 years, which was 10.9 years (median) after BC diagnosis. Sixty‐two percent of the cases were diagnosed with congestive HF, 19% with dilated cardiomyopathy, and 20% with both HF and dilated cardiomyopathy (online supplementary *Table* *S4*). Tumor characteristics can be found in online supplementary *Table* *S5*.

**Table 1:** Characteristics of heart failure cases and matched controls

	Cases (*n* = 102)	Controls (*n* = 306)	*P*‐value*
Age at breast cancer diagnosis^a^			
Median age (years)	51.1 [45.1–55.2]	51.1 [45.5–55.2]	
30–39 years	12 (11.8)	36 (11.8)	
40–49 years	31 (30.4)	93 (30.4)	
50–59 years	48 (47.1)	144 (47.1)	
60–65 years	11 (10.8)	33 (10.8)	
Year of breast cancer diagnosis^a^			
1976–1979	12 (11.8)	41 (13.4)	
1980–1989	26 (25.5)	83 (27.1)	
1990–1999	32 (31.4)	85 (27.8)	
2000–2009	32 (31.4)	97 (31.7)	
Type of surgery			
Breast conserving surgery	38 (37.3)	167 (54.6)	
Mastectomy	64 (62.8)	139 (45.4)	<0.001
Radiotherapy			
No	14 (13.7)	38 (12.4)	
Yes	88 (86.3)	268 (87.6)	0.75
Chemotherapy			
No chemotherapy	44 (43.1)	185 (60.5)	
Chemotherapy, no trastuzumab	44 (43.1)	111 (36.3)	
Chemotherapy and trastuzumab	14 (13.7)	10 (3.3)	
No chemotherapy, trastuzumab	0 (0)	0 (0)	<0.001
Endocrine therapy			
No	65 (63.7)	241 (78.8)	
Yes	37 (36.3)	65 (21.2)	0.002
Age at HF diagnosis/cut‐off date^b^			
Median age (years)	62.1 [53.8–69.2]	62.1 [53.8–69.2]	
<40 years	3 (2.9)	10 (3.3)	
40–49 years	13 (12.8)	38 (12.4)	
50–59 years	24 (23.5)	72 (23.5)	
60–69 years	37 (36.3)	111 (36.3)	
≥70 years	25 (24.5)	75 (24.5)	
Time to HF/cut‐off date^a^			
Median time (years)	10.9 [3.5–18.4]	10.9 [3.5–18.4]	
<1 year	6 (5.9)	18 (5.9)	
1–4 years	22 (21.6)	66 (21.6)	
5–9 years	17 (16.7)	51 (16.7)	
10–14 years	19 (18.6)	56 (18.3)	
15–19 years	18 (17.7)	55 (18.0)	
≥20 years	20 (19.6)	60 (19.6)	

Values are expressed as *n* (%), or median [interquartile range].

HF, heart failure; IQR, interquartile range.

a Matching factor for control selection.

b The variable ‘age at HF diagnosis/cut‐off date’ is derived from matching factors ‘age at breast cancer diagnosis’ and ‘time to HF/cut‐off date’. Cut‐off date was defined as date of HF diagnosis for cases, and for controls as the date of breast cancer plus the time interval between breast cancer and HF diagnosis for the corresponding case.

* *P*‐value for difference in non‐matching variables between cases and controls, calculated using a conditional model (accounting for matching variables).

### All treatments

In women treated with radiotherapy, median MHD was 6.8 Gy for cases and 3.9 Gy for controls. Considering simultaneously all the treatment types that patients had received (*Table* 2, model I), the HF RRs in MHD categories 2–9 and 10+ Gy were 0.8 (95% CI 0.49–1.3) and 1.2 (95% CI 0.72–1.9), compared to patients with a MHD of ≤2 Gy. Patients exposed to a low MHD were chosen as reference category instead of patients not treated with radiotherapy because this latter group might be a selective patient group. Patients who received anthracyclines but no trastuzumab had a 6.9 times increased HF rate (95% CI 3.5–13.6) compared to patients not treated with chemotherapy. After anthracyclines and trastuzumab, the HF RR was 34.9 (95% CI 11.1–110.1). Analysis excluding the six cases (and corresponding controls) treated with trastuzumab for whom recovery in the first year after HF diagnosis was unknown, showed a lower RR (17.5, 95% CI 5.0–61.3) for anthracyclines with trastuzumab vs. no chemotherapy (online supplementary *Table* *S6*). HF in women who received anthracyclines without trastuzumab occurred >10 years after diagnosis of BC in 21/33 cases while, in contrast, all 14 cases of failure who received trastuzumab occurred within the first 4 years after BC (online supplementary *Table* *S7*). In patients who received endocrine therapy with tamoxifen only, the RRs were not increased compared with patients who did not receive endocrine therapy but in the 13 patients treated with aromatase inhibitors only, a RR of 4.0 was recorded (95% CI 1.0–16.3, *P* = 0.06). Restricting analyses to patients without second malignancies did not materially affect any of the results (online supplementary *Table* *S6*).

**Table 2:** Associations between breast cancer treatment and heart failure risk

Total	Median value (IQR)	Cases (*n* = 102)	Controls (*n* = 306)	RR	Floating 95% CI	*P*‐value
**Model I: Model for all treatments considered simultaneously**						
Radiotherapy						
Median [IQR] mean heart dose^a^ (Gy)		6.8 [0.9–13.7]	3.9 [0.9–13.4]			
Mean heart dose 0–1 Gy	0.4 [0.2–0.9]	28 (27.5)	96 (31.4)	1.0^c^	0.53–1.9	
Mean heart dose 2–9 Gy	4.3 [3.8–6.6]	26 (25.5)	83 (27.1)	0.8	0.49–1.3	0.53
Mean heart dose ≥10 Gy	14.6 [13.6–17.0]	33 (32.4)	83( 27.1)	1.2	0.72–1.9	0.74
No radiotherapy		14 (13.7)	38 (12.4)	1.4	0.73–2.8	0.51
Mean heart dose unknown^b^		1 (0.9)	6 (2.0)	–	–	
Chemotherapy						
No chemotherapy		44 (43.1)	185 (60.5)	1.0^c^	0.60–1.7	
CMF‐like		9 (8.8)	48 (15.7)	0.7	0.30–1.5	0.35
Anthracyclines^e^		33 (32.4)	60 (19.6)	6.9	3.5–13.6	<0.001
Anthracyclines and trastuzumab^e^		14 (13.7)	7 (2.3)	34.9^f^	11.1–110.1	<0.001
Other type of chemotherapy or type unknown		2 (2.0)	6 (2.0)	–g	–	
Endocrine therapy						
No endocrine therapy		65 (63.7)	241 (80.4)	1.0^c^	0.67–1.5	
Tamoxifen		20 (19.6)	41 (11.6)	1.5	0.80–2.8	0.29
Tamoxifen and aromatase inhibitors		8 (7.8)	17 (6.0)	1.6	0.57–4.5	0.42
Aromatase inhibitors		8 (7.8)	5 (1.3)	4.0	1.0–16.3	0.06
Type of endocrine therapy unknown		1 (0.9)	2 (0.7)	–g	–	
**Model II** h: **Cumulative anthracycline dose by treatment with radiotherapy and trastuzumab** i						
No radiotherapy						
Median [IQR] cumulative anthracycline dose^j^ (mg/m^2^)		242 [230–302]	252 [241–302]			
Total		14	38			
No trastuzumab						
0 mg/m^2^		11 (78.6)	33 (86.8)	–	–	
≤240 mg/m^2^	231 [231–231]^k^	1 (7.1)	0 (0)	–g	–	
>240 mg/m^2^	252 [241–302]^k^	1 (7.1)	5 (13.2)	–g	–	
With trastuzumab						
>240 mg/m^2^	302 [302–302]^k^	1 (7.1)	0 (0)	–g	–	
Radiotherapy						
Median [IQR] cumulative anthracycline dose^j^ (mg/m^2^)		247 [240–319]	240 [240–300]			
Total		88	268			
No trastuzumab						
0 mg/m^2^		43 (48.9)	203 (75.8)	1.0^c^	0.48–2.1	
≤240 mg/m^2^	240 [221–240]^k^	9 (10.2)	26 (9.7)	3.3	1.5–7.2	0.02
>240 mg/m^2^	300 [252–360]^k^	23 (26.1)	29 (10.8)	8.6	4.7–15.6	<0.001
With trastuzumab						
≤240 mg/m^2^	240 [240–240]^k^	13 (14.8)	10 (3.7)	25.3	9.7–65.9	<0.001
**Model III** e **: Estimated mean heart dose by anthracycline and trastuzumab**						
No anthracyclines or trastuzumab						
Median [IQR] mean heart dose^a^^,^ b (Gy)		3.8 [0.2–14.2]	3.8 [0.4–14.2]			
Total		54	236			
0–1 Gy	0.4 [0.2–0.9]	9 (16.7)	60 (25.4)	1.0^c^^,^ d	0.43–2.3	
2–9 Gy	3.8 [3.8–5.4]	12 (22.2)	64 (27.1)	0.6	0.31–1.1	0.30
≥10 Gy	14.7 [14.2–18.0]	21 (38.9)	77 (32.6)	0.7	0.46–1.2	0.57
No radiotherapy		11 (20.4)	33 (14.0)	1.1	0.52–2.2	0.87
Mean heart dose unknown^b^		1 (1.9)	2 (0.9)	–g	–	
Anthracyclines but not trastuzumab						
Median [IQR] mean heart dose^a^^,^ b (Gy)		6.9 [0.9–12.0]	0.9 [0.2–6.3]			
Total		34	60			
0–1 Gy	0.9 [0.2–0.9]	9 (26.5)	29 (48.3)	1.0^c^	0.42–2.4	
2–9 Gy	6.4 [4.5–6.9]	13 (38.2)	17 (28.3)	1.2	0.55–2.8	0.70
≥10 Gy	15.2 [12.0–16.9]	10 (29.4)	5 (8.3)	2.8	0.89–9.0	0.15
No radiotherapy		2 (5.9)	5 (8.3)	1.2	0.27–5.6	0.82
Mean heart dose unknown^b^		0 (0)	4 (6.7)	–g	–	
Trastuzumab						
Median [IQR] mean heart dose^a^^,^ b (Gy)		0.9 [0.2–0.9]	0.9 [0.9–6.4]			
Total		14	10			
0–1 Gy		10 (71.4)	6 (60.0)	–	–	
2–9 Gy		2 (14.3)	3 (30.0)	–g	–	
≥10 Gy		1 (7.2)	1 (10.0)	–g	–	
No radiotherapy		1 (7.2)	0 (0)	–g	–	
**Model IV** l **: Joint effects of mean heart dose and anthracyclines**						
Mean heart dose <10 Gy, no anthracyclines	3.3 [0.4–3.9]	32 (31.4)	160 (52.3)	1.0^c^	0.66–1.5	
Mean heart dose ≥10 Gy, no anthracyclines	14.7 [14.2–18.0]	21 (20.6)	77 (25.2)	1.1	0.59–1.9	0.85
Mean heart dose <10 Gy, anthracyclines	0.9 [0.4–5.0]	37 (36.3)	57 (18.6)	6.3	3.0–13.2	<0.001
Mean heart dose ≥10 Gy, anthracyclines	14.9 [12.0–16.8]	11 (10.8)	6 (2.0)	12.4	4.0–39.2	<0.001
Mean heart dose unknown^b^		1 (1.0)	6 (2.0)	–g	–	–

CI, confidence interval; CMF, cyclophosphamide, methotrexate, 5‐fluorouracil; IQR, interquartile range; RR, rate ratio.

RRs for heart failure were estimated using logistic regression conditional on the matching variables.

a In patients treated with radiotherapy.

b Heart doses were unknown for seven patients (one case, six controls) because their radiotherapy charts were unavailable.

c Reference category. CI for categorical exposure variables were estimated for each category, including the reference category, from the amount of information in that category.

d *P* for trend across categories 0.48.

e Anthracycline treatment consisted of an epirubicin‐containing regimen for 14/48 cases and 16/67 controls, and of a doxorubicin‐containing regimen for 34/48 cases and 51/67 controls. Trastuzumab was mostly given in combination with anthracyclines.

f RR for anthracyclines plus trastuzumab vs. anthracyclines without trastuzumab is 5.5 (95% CI 1.9–16.0).

g Insufficient numbers to produce reliable estimates for this category.

h Model additionally included a dichotomous variable for endocrine therapy (no/yes).

i Range cumulative anthracycline dose was 90–612 mg/m^2^ doxorubicin equivalent, the commonest dose was four times 60 mg/m^2^. Two patients were treated with an anthracycline for breast cancer and then later retreated with an anthracycline for a recurrence or second malignancy. Cumulative anthracycline range for all other patients was 90–366 mg/m^2^. The commonest regimens were: AC (doxorubicin and cyclophosphamide), FAC (5‐fluorouracil, doxorubicin, cyclophosphamide), TAC (docetaxel, doxorubicin, cyclophosphamide), and FEC (5‐fluorouracil, epirubicin, cyclophosphamide).

j In patients treated with anthracyclines.

k Median anthracycline dose and IQR in patients treated with and without trastuzumab.

l Model also included dichotomous variables for radiotherapy (no/yes), trastuzumab (no/yes), and endocrine therapy (no/yes).

### Effect of anthracycline dose

**Figure 1:**
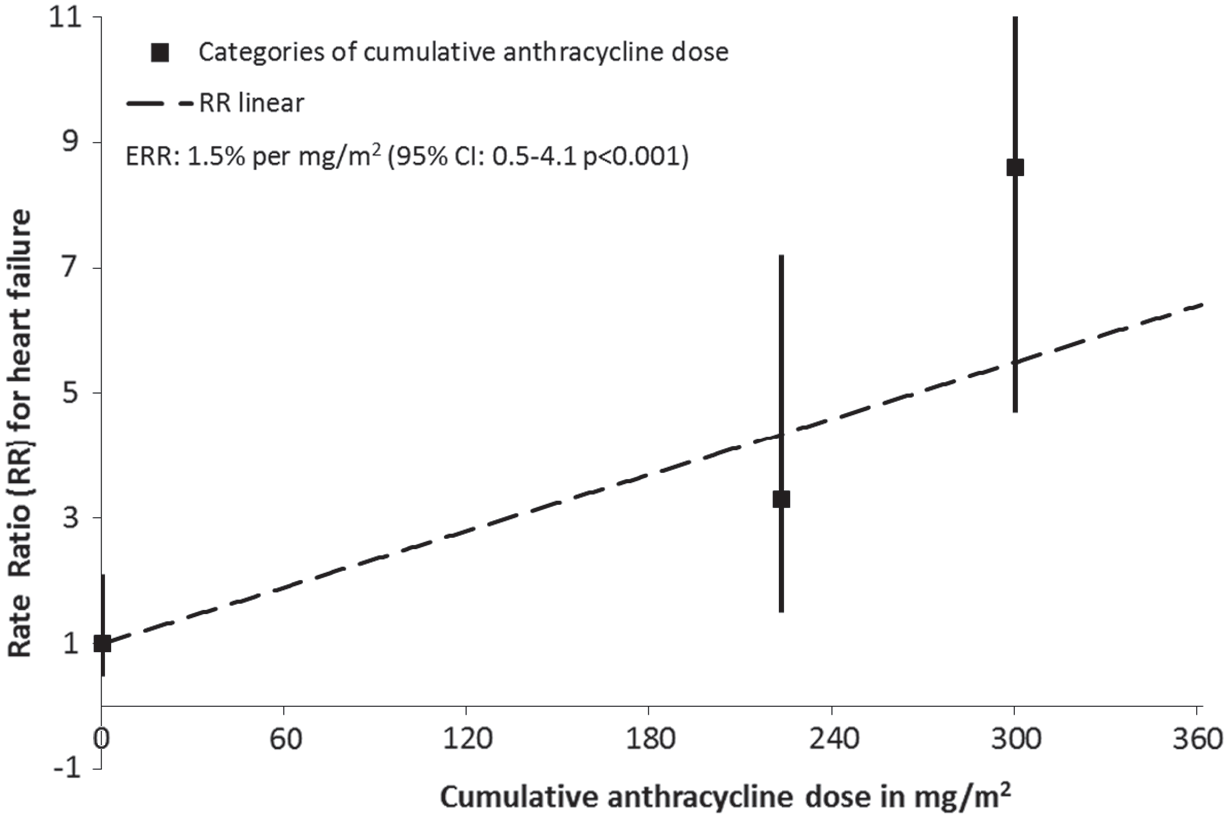
Excess rate ratio (ERR) in heart failure by cumulative anthracycline dose. The regression line is the best fitting linear dose–response relationship. This results in an ERR of 1.5% per mg/m^2^ [95% confidence interval (CI) 0.5–4.1]. Squares indicate point estimates for dose categories (no anthracycline‐based chemotherapy, ≤240 mg/m^2^ cumulative anthracycline dose, and >240 mg/m^2^ cumulative anthracycline dose, see *Table* 2) and are plotted at the mean cumulative anthracycline dose of each category. There was no significant departure from linearity observed. Patients treated with trastuzumab were excluded from this analysis.

Among patients who were not treated with radiotherapy, the effect of increasing anthracycline dose could not be assessed due to insufficient numbers (*Table* 2, model II). The HF rate increased with increasing anthracycline dose and, compared with patients who did not receive anthracyclines, the RR for patients with a median cumulative anthracycline dose of 240 mg/m^2^ (IQR 221–240) was 3.3 (95% CI 1.5–7.2) while for patients with a median cumulative anthracycline dose of 300 mg/m^2^ (IQR 252–360) the RR was 8.6 (95% CI 4.7–15.6). A linear dose–response relationship was observed, with HF rate increasing by 1.5% per mg/m^2^ anthracycline dose (95% CI 0.5–4.1) (*Figure* 1). Modelled 10‐year cumulative incidence was 1.4% for patients treated with a cumulative anthracycline dose of ≤240 mg/m^2^ and 3.1% for patients with a cumulative anthracycline dose of >240 mg/m^2^.

### Effect of heart radiation dose

Heart failure rate did not increase significantly with MHD. Compared to 0–1 Gy MHD, HF RRs were 0.6 (95% CI 0.31–1.1) for MHD 2–9 Gy and 0.7 (95% CI 0.46–1.2) for ≥10 Gy MHD (*Table* 2, model III). Similarly, in patients who received radiotherapy and anthracyclines but not trastuzumab, the HF rate did not increase significantly with increasing MHD. Compared with a baseline of 0–1 Gy MHD, HF RRs were 1.2 (95% CI 0.55–2.8) for MHD 2–9 Gy and 2.8 (95% CI 0.89–9.0) for ≥10 Gy MHD. The ERR based on a linear dose–response relationship for MHD in the entire study group was 1%/Gy increase (95% CI −2% to 10%). For patients treated with anthracyclines, a non‐significant increase of 8%/Gy increase in MHD was observed (95% CI −3% to 43%). For patients not treated with anthracyclines, no risk increase was seen (ERR = 0%/Gy 95% CI −3% to 8%). As for MHD, there were no significant associations between mean left ventricle dose and HF risk (online supplementary *Table* *S8*). A model assessing the joint effects of MHD and anthracyclines showed that, compared to women with a MHD <10 Gy and no anthracyclines, women with a MHD ≥10 Gy and no anthracycline had a HF RR of 1.1 (95% CI 0.59–1.9), women with a MHD <10 Gy and anthracyclines had a RR of 6.3 (95% CI 3.0–13.2) and women with a MHD ≥10 Gy and anthracyclines had a RR of 12.4 (95% CI 4.0–39.2) (*Table* 2, model IV).

Considering radiation dose–volume parameters in patients treated with anthracyclines without trastuzumab, there was no increased HF risk in patients according to increasing volume of the heart receiving 5 or 10 Gy (*Table* 3). However, in patients of whom ≥20% of the heart was exposed to 20 Gy (V_20_), the HF RR was 5.7 (95%CI 1.7–19.4) relative to women with V_20_ <10%. Also, in patients with 10–19% of the heart exposed to 25 Gy (V_25_) and 8 cases with V_25_ ≥20%, HF RRs were 4.1 (95% CI 1.0–15.9) and 7.8 (95% CI 1.8–34.6), respectively, relative to baseline (V_25_ <10%).

**Table 3:** Associations between percentage of heart volume receiving ≥5 to ≥25 Gray (V_5_ to V_25_) and heart failure risk in patients treated with anthracyclines and without trastuzumab

Percentage of heart volume receiving 5–25 Gy (V_5_ to V_25_)	Cases (*n* = 29)^a^	Controls (*n* = 51)^a^	RR	Floating 95% CI	*P*‐value
V_5_					
Median value [IQR]	35.4% [11.2–67.1]	3.0% [0–43.7]			
0–9%	6 (20.7)	28 (54.9)	1.0^b^	0.31–3.3	
10–19%	4 (13.8)	5 (9.8)	1.1	0.29–4.3	0.90
≥20%	19 (65.5)	18 (35.3)	2.7	1.2–6.2	0.11
V_10_					
Median value [IQR]	30.7% [7.0–59.2]	1.7% [0–38.5]			
0–9%	10 (34.5)	32 (62.8)	1.0^b^	0.40–2.5	
10–19%	3 (10.3)	4 (7.8)	0.7	0.15–3.6	0.74
≥20%	16 (55.2)	15 (29.4)	2.8	1.1–7.0	0.06
V_20_					
Median value [IQR]	15.0% [3.2–26.0]	1.1% [0–13.8]			
0–9%	13 (44.8)	36 (70.6)	1.0^b^	0.43–2.3	
10–19%	4 (13.8)	10 (19.6)	1.4	0.36–5.3	0.65
≥20%	12 (41.4)	5 (9.8)	5.7	1.7–19.4	0.01
V_25_					
Median value [IQR]	11.2% [2.0–20.1]	1.0% [0.1–8.9]			
0–9%	14 (48.3)	43 (84.3)	1.0^b^	0.43–2.3	
10–19%	7 (24.1)	5 (9.8)	4.1	1.0–15.9	0.05
≥20%	8 (27.6)	3 (5.9)	7.8	1.8–34.6	0.01

CI, confidence interval; IQR, interquartile range; RR, rate ratio; V_5_‐V_25_, dose–volume parameters.

a Dose–volume estimation was not possible for the three patients treated with anthracyclines and without trastuzumab, all cases, who had been treated with a combination of orthovoltage and electron/megavoltage radiotherapy, and for the four patients treated with anthracyclines without trastuzumab, all controls, for whom the radiotherapy charts were unavailable.

b Reference category.

### Cardiac risk factors

There was some evidence that obese women (BMI ≥30 kg/m^2^) had an increased HF rate compared to women with a BMI <25 kg/m^2^ (RR 2.4, 95% CI 1.0–5.4, *P* = 0.07) and that perimenopausal women had an increased rate compared to premenopausal women (RR 2.2, 95% CI 1.0–4.8, *P* = 0.10) (online supplementary *Table* *S9*). No significant associations were found between HF rate and smoking or known co‐morbidities.

## Discussion

This is the first study to assess the relationships between radiation dose to the heart, anthracycline dose and the risk of subsequent HF in women treated for early BC. There was no significant association between MHD and HF rate or between mean left ventricle dose and HF rate. In contrast, there was a strong, dose‐dependent, relation between HF rate and anthracycline dose. The rate was increased nearly nine‐fold for patients who received a cumulative dose of >240 mg/m^2^ doxorubicin equivalent. Also, in patients treated with both anthracyclines and radiotherapy of whom ≥20% of the heart was exposed to 20 Gy (V_20_), the HF rate was significantly increased relative to other women whose V_20_ was <10%, suggesting that anthracycline‐related cardiotoxicity may be exacerbated by irradiating a large volume of the heart to a high dose. This latter finding is, however, based on only 17 patients, and so needs confirmation in other studies. Although our study included only 24 patients treated with trastuzumab, a strongly increased HF risk was observed for patients receiving trastuzumab compared with those who did not receive it. There was a borderline significant association between treatment with aromatase inhibitors and HF rate, based on 13 patients, but no significant increases associated with other types of endocrine therapy.

In the Netherlands, as in many countries, national BC guidelines recommend that women at high BC recurrence risk should be considered for treatment with anthracycline‐based chemotherapy and, if they are HER2 positive, with 1 year of trastuzumab. Our results are, therefore, particularly relevant to women at high risk of recurrence. It is, however, important to note that HF rates in the general population of women in the age range included in the current study are quite low.^26^ Therefore, these large proportional increases do not result in a large absolute increase in HF rates for BC patients, at least not in the first 10–15 years after treatment. Although the absolute HF rates in young patients may still be low, recently published mortality rates^27^ indicate that HF preceded by anthracycline‐based chemotherapy is a disease with a poor prognosis that requires early recognition, and studies investigating (medical) interventions are needed.

The time between BC diagnosis and HF diagnosis was much shorter in cases treated with trastuzumab than in cases treated with anthracyclines but not trastuzumab. This difference occurred despite exclusion of reversible treatment‐related HF cases occurring within 1 year of treatment. The short latency between trastuzumab exposure and development of HF has been reported in large randomized trials in which trastuzumab‐related HF developed during or a few months after treatment and recovered within 6 years of stopping trastuzumab in >60% of patients.^7^, ^8^ It is likely that the presentation of HF differed according to the exposure. HF diagnoses in patients treated with trastuzumab may have been less severe given that patients on trastuzumab undergo repeated surveillance during treatment, whereas patients treated with anthracyclines without trastuzumab only undergo (repeated) echocardiography if they present with symptoms. The absence of cardiac surveillance in this group possibly resulted in a late HF diagnosis, since a large prospective echocardiographic monitoring study by Cardinale *et al*.^19^ showed that most cardiotoxicity events occurred within the first year after anthracycline‐containing chemotherapy. Other studies have observed increased HF rates after anthracyclines^13^, ^22^, ^23^ and after trastuzumab treatment^20^, ^21^, ^28^ and have shown these differences in time dependence and presentation. The numbers in the other studies are, however, lower than in our study.

The radiotherapy results in this nested case–control study differ somewhat from those reported in our cohort study,^2^ which reported increased HF rates after radiotherapy without anthracycline treatment. For the current case–control study more detailed information was collected on HF diagnosis and recovery, and the HF definition was stricter. Interestingly, Saiki *et al*.^3^ also observed an increased rate of HF after radiotherapy for BC, which was restricted to HF with a preserved EF (HFpEF) (odds ratio 5.2 per log MHD, 95% CI 1.4–19.1) while no increased risk was seen for HFrEF (odds ratio 1.2 per log MHD, 95% CI 0.22–6.0). Exclusion of cases with HFpEF in the current case–control study may explain why we did not observe a clearly increased HF rate after radiotherapy.

The increased HF rate observed for aromatase inhibitor use only is intriguing, but should interpreted with caution as we were unable to stratify our analyses for menopausal status due to limited patient numbers (these findings were based on 13 patients). Women treated with aromatase inhibitors only are generally postmenopausal and may have, due to age and menopausal status, a worse cardiovascular risk profile, irrespective of type of endocrine therapy. However, there was no evidence that menopausal status was a confounder of the relationship between aromatase inhibitors and HF (data not shown). A number of studies have suggested that aromatase inhibitors may increase the HF risk.^29^, ^30^ Haque *et al*.^29^ studied CVD outcomes in over 13 000 post‐menopausal BC patients and found a higher HF risk in patients treated with aromatase inhibitors compared to patients treated with tamoxifen only (hazard ratios 1.1–1.3). The fact that we did not observe an increased HF rate in patients treated with both aromatase inhibitors and tamoxifen may be explained by treatment duration; upfront aromatase inhibitors were generally prescribed for a duration of 5 years compared to a duration of 2.5 years when combined with tamoxifen (Dutch treatment guidelines, http://www.oncoline.nl).

Strengths of our study include comprehensive data collection from medical files, including detailed treatment information, with individual cumulative anthracycline dose and estimated individual patient radiation MHD, mean left ventricle dose, and dose–volume parameters. HF diagnoses were based on information from both a general practitioner and a cardiologist. Information on the presence of cardiovascular risk factors at BC diagnosis was collected. The strict exclusion criteria that we applied with regard to other cancer treatments and history of clinically significant CVD eliminated their influence on the risk estimates. Exclusion of these cases, however, also means that it was not possible to assess the effect of BC treatments on HF rates in patients with a history of CVD before HF diagnosis. Furthermore, patient numbers in this study were too small to assess interactions between cardiotoxic treatment and cardiovascular risk factors or menopausal status. Another potential limitation is the inaccurate registration of duration of endocrine treatment in the medical files, and the incompleteness of detailed cardiac information, such as EF measurements.

In conclusion, our results show a clear dose‐dependent increase in HF risk after anthracycline‐based chemotherapy. In the absence of anthracycline‐based chemotherapy, our data suggest that modern BC radiotherapy does not increase HF risk. HF risks associated with high doses to the heart (i.e. MHDs exceeding 10 Gy or large volumes of the heart exposed to ≥20 Gy) in patients receiving anthracycline‐based chemotherapy require further studies. Treatment with trastuzumab increases HF risk within 1–2 years of treatment and anthracycline‐based chemotherapy increases HF risk in the first 10 years after treatment. Both anthracyclines and trastuzumab cure many women of their cancers, and the absolute gain for a typical woman selected to receive chemotherapy is usually >5% reduction in BC mortality risk over the next 10 years. For most women this is likely to substantially exceed treatment‐related HF risk. However, our results do emphasize the need to support ongoing efforts to evaluate preventative strategies.

### Funding

This work was supported by the Dutch Cancer Society (grant number NKI 2008‐3994) and Pink Ribbon (grant number 2012.WO39.C143) FD, CT, and SD received funding from Cancer Research UK (grant number C8225/A21133), the British Heart Foundation Centre for Research Excellence, Oxford (grant number RE/13/1/30181) as well as core funding from Cancer Research UK, the UK Medical Research Council and the British Heart Foundation to the Oxford University Clinical trial Service Unit (grant number MC_U137686858).

**Conflict of interest:** G.S.S.: institutional research support from Roche; modest. All other authors have nothing to disclose.

## Supporting information


**Methods S1.** Details of the data collection procedures and the eligibility criteria for the cohort.
**Methods S2.** Grading according to adaptation of the National Cancer Institute Common Terminology Criteria for Adverse Events versions 3.0 and 4.0.
**Methods S3.** Radiation dosimetry.
**Table S1.** Radiotherapy techniques received by 408 women with breast cancer at the Netherlands Cancer Institute or the Erasmus MC Cancer Institute in the Netherlands during 1970 to 2009.
**Table S2.** Reasons for exclusion of cases.
**Table S3.** Characteristics of cases and controls by chemotherapy and trastuzumab treatment
**Table S4.** Characteristics of included cases
**Table S5.** Breast cancer characteristics of heart failure (HF) cases and matched controls
**Table S6.** Additional analyses on associations between breast cancer treatment and heart failure (HF) risk with restrictions on (I) cases with unknown recovery status 1 year after diagnosis of HF, (II) cases treated with trastuzumab, and (III) patients with a second malignancy
**Table S7.** Time between breast cancer diagnosis and heart failure (HF) diagnosis by type of chemotherapy and trastuzumab treatment
**Table S8.** Associations between estimated mean left ventricular dose and heart failure (HF) risk
**Table S9.** Associations between patient‐related risk factors at breast cancer diagnosis and heart failure risk
