# Supplementary material for: Heart failure after treatment for breast cancer
Source: Eur J Heart Fail. 2019 Nov 12;22(2):366–74. doi: 10.1002/ejhf.1620 (PMC7137787; doi:10.1002/ejhf.1620)
Supplement: Supplementary file 1 — Methods S1. Details of the data collection procedures and the eligibility criteria for the cohort. Methods S2. Grading according to adaptation of the National Cancer Institute Common Terminology Criteria for Adverse Events versions 3.0 and 4.0. Methods S3. Radiation dosimetry. Table S1. Radiotherapy techniques received by 408 women with breast cancer at the Netherlands Cancer Institute or the Erasmus MC Cancer Institute in the Netherlands during 1970 to 2009. Table S2. Reasons for exclusion of cases. Table S3. Characteristics of cases and controls by chemotherapy and trastuzumab treatment Table S4. Characteristics of included cases Table S5. Breast cancer characteristics of heart failure (HF) cases and matched controls Table S6. Additional analyses on associations between breast cancer treatment and heart failure (HF) risk with restrictions on (I) cases with unknown recovery status 1 year after diagnosis of HF, (II) cases treated with trastuzumab, and (III) patients with a second malignancy Table S7. Time between breast cancer diagnosis and heart failure (HF) diagnosis by type of chemotherapy and trastuzumab treatment Table S8. Associations between estimated mean left ventricular dose and heart failure (HF) risk Table S9. Associations between patient‐related risk factors at breast cancer diagnosis and heart failure risk [file EJHF-22-366-s001.docx]

**Supplemental material – ONLINE ONLY**

**Supplemental methods 1. Details of the data collection procedures and the eligibility criteria for the cohort**

Female breast cancer patients (stage I-IIIA or ductal carcinoma in situ [DCIS]) were selected from the hospital-based registries of the Netherlands Cancer Institute, Amsterdam or the Erasmus MC - Cancer Institute, Rotterdam, the Netherlands. All patients had to have received at least surgery. Patients who had been treated with radiation therapy between the diaphragm and the chin, or treated with any type of chemotherapy before breast cancer diagnosis were not included in the cohort. Data collection from the registries and medical files included the following variables: date of birth, breast cancer diagnosis, tumour histology, stage, type of surgery, radiation fields, chemotherapy regimen, endocrine treatment, date of first recurrence and distant metastasis, date, diagnosis and treatment of previous and subsequent malignancies, history of CVD before breast cancer diagnosis, dates and diagnoses of cardiovascular events, smoking, hypertension, diabetes mellitus, hypercholesterolemia, date of last known medical status, and cause of death (according to the International Classification for Diseases, 10th revision).

Because data collection on cardiovascular disease incidence through questionnaires to general practitioners and cardiologists is very labour intensive, and because we were interested in long-term cardiovascular disease risks following radiation therapy and chemotherapy, the current study was restricted to patients who were diagnosed with breast cancer before the age of 65 years (in the study period very few patients older than 65 years received chemotherapy). When the first part of the cohort was established in the 1990s, it was generally assumed that increased cardiovascular risks did not emerge until the second decade after breast cancer treatment. Hence, for patients diagnosed between 1970 and 1986, cardiovascular disease information was acquired only for ten-year survivors(1). In addition, because during this period of diagnosis the majority of patients was treated with surgery plus radiation therapy, for reasons of efficiency, a stratified sample was taken of all patients treated with surgery plus radiation therapy, stratified by age. For all other treatment combinations, all ten-year survivors were included in the study. When the cohort was expanded with patients diagnosed between 1987 and 2009, we were also interested in the possibly increased cardiovascular disease risks after anthracyclines, which were thought to occur earlier after breast cancer treatment than radiation therapy effects. For patients diagnosed between 1987 and 2009, we therefore aimed to collect cardiovascular disease information for all one-year survivors. However, because funding resources were limited, we were obliged to make a patient selection for part of the expansion; the years of diagnosis 1994 to 2000. For patients diagnosed during this period we again took a stratified sample of all patients treated with surgery plus radiation therapy, stratified by age. For other treatment combinations, all one-year survivors were included. For the years of diagnosis 1987 to 1993 and 2001 to 2009 all one-year survivors were included. Selection of patients was always random within each age stratum and by definition independent of cardiovascular disease diagnosis, as the hospital-based registries did not contain any data on cardiovascular disease.

To complete cardiovascular follow-up in the entire cohort, approximately 10,000 letters were sent to general practitioners and cardiologists. In The Netherlands, all residents are expected to have a general practitioner. Medical correspondence from attending physicians is sent to the general practitioner. Records are preserved by the general practitioner throughout a patient’s life and for at least 15 years after a patient’s death. For patients treated before 2000, complete follow-up information to at least January 1, 2009 was available for 82% of the study cohort. For patients treated in 2000-2009, complete follow-up information through at least January 1, 2012, was available for 71% of the study cohort. For the other patients, the current/last general practitioner was unknown to us or unwilling to participate in the study. Selection bias introduced by general practitioners is unlikely as patient information did not appear to play a role in the general practitioner´s decision to return the questionnaires; for 61% of the patients with incomplete information, the GP was unknown or did not respond to any of the questionnaires. Less than 1% of patients were lost to follow-up because their medical files had been destroyed. Median follow-up duration was 14 years for the entire cohort; 23 years for patients diagnosed with breast cancer ≤1986 and 12 years for patients diagnosed >1986.

**References**

1. Hooning MJ, Botma A, Aleman BM, Baaijens MH, Bartelink H, Klijn JG, Taylor CW, Van Leeuwen FE. Long-term risk of cardiovascular disease in 10-year survivors of breast cancer. J Natl Cancer Inst. 2007;99(5):365-75.

**Supplemental methods 2. Grading according to adaptation of the National Cancer Institute Common Terminology Criteria for Adverse Events versions 3.0 and 4.0**

Cardiomyopathy and Heart Failure:

Grade 1 – Asymptomatic. Fractional shortening 30-24%, other cardiac myocardial imaging abnormalities less than those described below (e.g. mention of subjective hypokinesis on ECHO), or laboratory findings suggestive of myocardial dysfunction (e.g. elevated BNP)

Grade 2 – Ejection fraction decreased to 50-40% or a 10-19% drop from baseline; fractional shortening of 24-15%; symptoms with mild to moderate activity or exertion (1, 2)

Grade 3 – Ejection fraction decreased to 20-39% or a >20% drop from baseline; fractional shortening of <15%; symptoms at rest or with minimal activity or exertion; intervention indicated (e.g. drug treatment) but responsive to intervention

Grade 4 – Ejection fraction decreased <20%; refractory or poorly controlled heart failure requiring urgent intervention (e.g. continuous IV therapy, mechanical hemodynamic support or transplantation)

Grade 5 – Death

**References**

1. Cardinale D, Colombo A, Bacchiani G, Tedeschi I, Meroni CA, Veglia F, Civelli M, Lamantia G, Colombo N, Curigliano G, Fiorentini C, Cipolla CM. Early detection of anthracycline cardiotoxicity and improvement with heart failure therapy. Circulation. 2015;131(22):1981-8.

2. Schneider BP, O'Neill A, Shen F, Sledge GW, Thor AD, Kahanic SP, Zander PJ, Davidson NE. Pilot trial of paclitaxel-trastuzumab adjuvant therapy for early stage breast cancer: a trial of the ECOG-ACRIN cancer research group (E2198). British journal of cancer. 2015;113(12):1651-7.

**Supplemental methods 3. Radiation dosimetry**

Cardiac doses were estimated retrospectively by two of the authors (F.K.D. and C.W.T.). Retrospective estimation was necessary because CT-planning was used in the participating hospitals only from 2005 and so was not available for almost all study participants. Nor was individual anatomical information available.

*Chart categorization*

Regimens were identified from the radiotherapy charts of all 408 women in the study. The charts included diagrams or photographs of the treatment fields, and sometimes dose-plans. Details on surgery, target definition, field borders, target dose, applied total dose, dose per fraction, beam energy and use of shielding, wedges and bolus were collected. In total, 45 radiotherapy regimens were received by the 408 women in the study, 24 regimens for left-sided and 21 for right-sided breast cancer (Supplemental table 1). These regimens were further categorized into five technique categories including: tangential fields (42%, n=170), megavoltage internal mammary chain (IMC) fields (29%, n=118), brachytherapy (<1%, n=1), orthovoltage or mixed orthovoltage/megavoltage IMC fields (17%, n=71), and electron chest wall or IMC fields (12%, n=48).

*Selection of a typical CT scan*

Ten radiotherapy CT-planning scans were randomly selected from women referred for breast cancer radiotherapy in 2010. The treatment position was supine, with both arms above the head. CT slice thickness was 3mm. The whole heart was contoured including the pericardial sac extending superiorly to the inferior aspect of the pulmonary artery. The left ventricle was contoured using a published atlas^1^. To simulate mastectomy, the breast was virtually removed. To select a “typical CT-scan, first the most commonly used left-sided regimen was identified from the charts. This was a midline tangential regimen consisting of two opposing beams with alignment of the posterior borders, used in the 1980s up to the early 2000s. Second, the regimen was reconstructed on each of the ten CT scans and mean whole heart doses (MHDs) were reviewed. Third, anatomical features which may influence cardiac doses from breast cancer radiotherapy were measured, including: sternal length, heart volume, chest wall separation distance and the Haller index (ratio of height between the anterior spine and posterior sternum to the transverse width of the heart). A ‘typical CT-scan’, which was not atypical for any of the anatomical factors reviewed, was then selected as the scan with a MHD closest to average (MHD “typical CT-scan”: 4.8 Gy, average MHD ten CT scans: 4.7 Gy (range 1.9-9.1 Gy)).

*Regimen reconstruction*

All other regimens identified from the radiotherapy charts were then reconstructed on the “typical CT-scan”. Dose-distributions were generated for cobalt, electron and megavoltage beams using a 3-dimensional CT treatment planning system (Varian Eclipse^TM^ version 10.0.39). The analytical anisotropic algorithm was used to calculate cardiac doses for photon plans, Monte Carlo was used for electron plans, and pencil beam for cobalt plans. Dose distributions from orthovoltage fields were generated using manual planning. The mean heart dose (MHD), mean left ventricle dose (MLVD), and the percent volume of heart receiving ≥5, ≥10, ≥20, and ≥25 Gy (V_5_ to V_25_) were estimated using dose volume histograms.

*Allocation of doses to individual women*

Doses were estimated for each woman included in the study using the total dose (100%) received as recorded from each individual radiotherapy chart and the dose volume histogram of the regimen received.

*Limitations*

The cardiac doses estimated are subject to several sources of uncertainty. Many of these uncertainties are common to all radiotherapy CT-planning studies and include delineation error, dose calculation algorithm error, set-up error, and cardiac and respiratory motion during treatment. In this study of women for whom no anatomical information is available another important source of uncertainty is inter-patient differences in anatomy. For left tangential radiotherapy inter-patient dose variability was assessed by reconstructing midline tangents on ten CT scans. The difference between the highest and lowest mean doses estimated for the whole heart and left ventricle (LV) was 7.2 Gy and 10.7 Gy respectively. For right tangential radiotherapy inter-patient dose variability was assessed by reconstructing fields on five of the ten CT-scans. The inter-patient dose variability for the whole heart and LV was 0.5 Gy and 0.2 Gy respectively.

***References***

1. Duane F, Aznar M, Bartlett F etal. A cardiac contouring atlas for radiotherapy. Radiother Oncol 2017; 122: 416-22

**Supplemental table 1. Radiotherapy techniques received by 408 women with breast cancer at the Netherlands Cancer Institute or the Erasmus MC Cancer Institute in the Netherlands during 1970-2009.**

**Supplemental table 2. Reasons for exclusion of cases**

| Characteristic | No. of patients | Left | Right |
| --- | --- | --- | --- |
| Total number of HF cases | 342 | 173 | 169 |
| Total number of included cases | 102 | 41 | 61 |
| Total number of excluded cases | 240 | 132 | 108 |
| Reason for exclusion |  |  |  |

| Ductal carcinoma in situ only | 9 | 6 | 3 |
| --- | --- | --- | --- |
| Age at breast cancer diagnosis >65 years | 9 | 7 | 2 |
| Year of breast cancer diagnosis <1976 | 74 | 36 | 38 |
| Recurrence^*^, metastasis or 2^nd^ cancer^†^ occurred before HF | 52 | 27 | 25 |
| Other cardiovascular diagnosis before HF^‡^ | 46 | 24 | 22 |
| HF diagnosis rejected by cardiologist | 11 | 7 | 4 |
| HF before breast cancer diagnosis | 5 | 2 | 3 |
| LVEF recovery (≥50%) within one year | 3 | 3 | 0 |
| Other^§^ | 14 | 9 | 5 |
| Unknown | 17 | 11 | 6 |

Abbreviations: HF, heart failure; LVEF, left ventricular ejection fraction.

^*^ (Loco)regional recurrence treated with radiotherapy

^†^ Second cancer treated with radiotherapy above the diaphragm

^‡^ Myocardial infarction (n=13), valvular dysfunction (n=20), or clinically significant heart disease before HF and after breast cancer diagnosis

^§^ Mostly administrative reasons, such as medical record lost

**Supplemental table 3. Characteristics of cases and controls by chemotherapy and trastuzumab treatment**

| **Characteristics** | **Chemotherapy** | | |  |  |  |
| --- | --- | --- | --- | --- | --- | --- |
|  | **No chemotherapy** | | **Chemotherapy, no trastuzumab** | | **Chemotherapy and trastuzumab** | |
|  | **N** | **%** | **N** | **%** | **N** | **%** |
| **Age at breast cancer diagnosis** |  |  |  |  |  |  |
| 30-39 years | 12 | 5.2 | 31 | 20.0 | 5 | 20.8 |
| 40-49 years | 60 | 26.2 | 59 | 38.1 | 5 | 20.8 |
| 50-59 years | 119 | 52.0 | 59 | 38.1 | 14 | 58.3 |
| 60-65 years | 38 | 16.6 | 6 | 3.9 | 0 | 0.0 |
| **Year of breast cancer diagnosis** |  |  |  |  |  |  |
| 1976-1979 | 43 | 18.8 | 10 | 6.5 | 0 | 0.0 |
| 1980-1989 | 81 | 35.4 | 28 | 18.1 | 0 | 0.0 |
| 1990-1999 | 60 | 26.2 | 57 | 36.8 | 0 | 0.0 |
| 2000-2009 | 45 | 19.7 | 60 | 38.7 | 24 | 100.0 |
| **Mean heart dose** |  |  |  |  |  |  |
| Mean heart dose 0-1 Gy | 57 | 24.9 | 50 | 32.3 | 16 | 66.7 |
| Mean heart dose 2-9 Gy | 68 | 29.7 | 38 | 24.5 | 5 | 20.8 |
| Mean heart dose ≥10 Gy | 58 | 25.3 | 55 | 35.5 | 2 | 8.3 |
| No radiotherapy | 44 | 19.2 | 7 | 4.5 | 1 | 4.2 |
| Mean heart dose unknown^†^ | 2 | 0.9 | 5 | 3.2 | 0 | 0.0 |
| **Smoking** |  |  |  |  |  |  |
| Not smoking at breast cancer diagnosis | 14 | 6.1 | 10 | 6.5 | 1 | 4.2 |
| Smoking at breast cancer diagnosis | 88 | 38.4 | 62 | 40.0 | 11 | 45.8 |
| Unknown | 127 | 55.5 | 83 | 53.6 | 12 | 50.0 |
| **Body mass index** |  |  |  |  |  |  |
| Normal weight (BMI<25) | 89 | 38.9 | 79 | 51.0 | 12 | 50.0 |
| Overweight (BMI 25-29) | 47 | 20.5 | 35 | 22.6 | 5 | 20.8 |
| Obese (BMI ≥30) | 15 | 6.6 | 9 | 5.8 | 3 | 12.5 |
| Unknown | 78 | 34.1 | 32 | 20.7 | 4 | 18.7 |
| **Comorbidity** |  |  |  |  |  |  |
| No diabetes | 227 | 99.1 | 152 | 98.1 | 24 | 100.0 |
| Diabetes | 2 | 0.9 | 3 | 1.9 | 0 | 0.0 |
|  |  |  |  |  |  |  |
| No hypertension | 215 | 93.9 | 149 | 96.1 | 24 | 100.0 |
| Hypertension | 14 | 6.1 | 6 | 3.9 | 0 | 0.0 |
|  |  |  |  |  |  |  |
| No AP, COPD and CVA | 227 | 99.1 | 153 | 98.7 | 24 | 100.0 |
| AP, COPD or CVA | 2 | 0.9 | 2 | 1.3 | 0 | 0.0 |
| **Menopausal** **status** |  |  |  |  |  |  |
| Premenopausal | 77 | 33.6 | 98 | 63.2 | 12 | 50.0 |
| Perimenopausal | 20 | 8.7 | 11 | 7.1 | 1 | 4.2 |
| Postmenopausal | 107 | 46.7 | 29 | 18.7 | 7 | 29.2 |
| Unknown | 25 | 10.9 | 17 | 11.0 | 4 | 16.7 |

Abbreviations: BMI, body mass index; AP, angina pectoris; COPD, chronic obstructive pulmonary disease; CVA, cerebrovascular accident.

^†^ Heart doses were unknown for 7 patients (1 case, 6 controls) because their radiotherapy charts were unavailable

| **Supplemental table 4. Characteristics of included cases** | |  | |  | |  |
| --- | --- | --- | --- | --- | --- | --- |
| Characteristic | No. of patients | | Left | | Right | |
| Total number of cases | 102 | | 41 | | 61 | |
| Confirmed by |  | |  | |  | |
| Cardiologist questionnaire/correspondence | 60 | | 20 | | 40 | |
| Correspondence in the medical record | 21 | | 10 | | 11 | |
| General practitioner only | 21 | | 11 | | 10 | |
| Event |  | |  | |  | |
| Congestive heart failure | 63 | | 27 | | 36 | |
| Cardiomyopathy | 19 | | 6 | | 13 | |
| Congestive heart failure & cardiomyopathy | 20 | | 8 | | 12 | |
| Left ventricular function^*^ |  | |  | |  | |
| Heart failure with reduced ejection fraction (<50%) | 25 | | 11 | | 14 | |
| 40-49% | 6 | | 3 | | 3 | |
| 30-39% | 5 | | 3 | | 2 | |
| <30% | 8 | | 4 | | 4 | |
| Unknown | 6 | | 1 | | 5 | |
| Heart failure with preserved ejection fraction (≥50%) | 2 | | 1 | | 1 | |
| Unknown^†^ | 54 | | 15 | | 25 | |
| Cardiomyopathy type^‡^ |  | |  | |  | |
| Dilated cardiomyopathy | 6 | | 2 | | 4 | |
| Restrictive cardiomyopathy | 0 | | 0 | | 0 | |
| Unknown^§^ | 28 | | 9 | | 19 | |
| LVEF recovery in patients treated with trastuzumab^\|\|^ |  | |  | |  | |
| Recovery LVEF without medication^¶^ | 2 | | 0 | | 2 | |
| Recovery LVEF with medication | 3 | | 2 | | 1 | |
| Recovery unknown, last LVEF <50% | 6 | | 3 | | 3 | |
| Recovery unknown | 3 | | 1 | | 2 | |
| History of CVD before breast cancer |  | |  | |  | |
| Conduction abnormality | 2 | | 0 | | 2 | |
| Ischemic heart disease | 0 | | 0 | | 0 | |
| History of CVD after breast cancer and before HF |  | |  | |  | |
| Conduction abnormality | 2 | | 0 | | 2 | |
| Ischemic heart disease | 2 | | 1 | | 1 | |
| Status at end of follow-up |  | |  | |  | |
| Alive | 71 | | 26 | | 45 | |
| Deceased, cardiac cause of death | 6 | | 2 | | 4 | |
| Deceased, other cause of death | 5 | | 4 | | 1 | |
| Deceased, unknown cause of death | 20 | | 9 | | 11 | |

Abbreviations: LVEF, left ventricular ejection fraction; CVD, cardiovascular disease; HF, heart failure.

^*^For the 81 cases with confirmation by cardiologist or correspondence in the medical record. Patients with an unknown left ventricular function included e.g. cases for which the cardiologist confirmed congestive heart failure but did not provide ejection fraction information, but also confirmed cardiomyopathy cases.

^†^No information on left ventricular function was available.

^‡^Cardiomyopathy cases which were confirmed by cardiologist and/or correspondence in the medical record.

^§^No information on cardiomyopathy type was available.

^||^ In total 14 cases had been treated with trastuzumab. Left ventricular function was available from echocardiogram reports retrieved through the medical records or correspondence with a cardiologist.

^¶^Left ventricular ejection fraction recovery (≥50%) which occurred ≥ 1 year after heart failure diagnosis.

**Supplemental table 5. Breast cancer characteristics of heart failure cases and matched controls**

|  | **Cases** |  | **Controls** |  | ***p^*^*** |
| --- | --- | --- | --- | --- | --- |
| Total | 102 (N) | 100 (%) | 306 (N) | 100 (%) |  |
| Laterality of breast cancer^†^ |  |  |  |  |  |
| Right | 61 | 59.8 | 133 | 43.5 |  |
| Left | 41 | 40.5 | 173 | 56.5 | *0.01* |
| Nodal status |  |  |  |  |  |
| Negative | 41 | 40.2 | 169 | 55.2 |  |
| Positive | 59 | 57.8 | 134 | 43.8 |  |
| Unknown | 2 | 2.0 | 3 | 1.0 | *0.01* |
| Tumor size |  |  |  |  |  |
| <2cm | 31 | 30.4 | 132 | 43.1 |  |
| 2-5cm | 51 | 50.0 | 124 | 40.5 |  |
| ≥5cm | 12 | 11.8 | 18 | 5.9 |  |
| Unknown | 8 | 7.8 | 32 | 10.5 | *0.01* |
| Breast cancer stage |  |  |  |  |  |
| Stage I | 21 | 20.6 | 93 | 30.4 |  |
| Stage II | 67 | 65.7 | 179 | 58.5 |  |
| Stage III | 9 | 8.8 | 15 | 4.9 |  |
| Unknown | 5 | 4.9 | 19 | 6.2 | *0.15* |

^*^ P-value for difference in non-matching variables between cases and controls, calculated using a conditional model (accounting for matching variables)

^†^ Although the imbalance in laterality was not seen the cohort in which the current study is nested, no apparent reason could be found in the exclusion of cases (see Supplemental table 2)

**Supplemental Table 6. Additional analyses on associations between breast cancer treatment and heart failure risk with restrictions on (I) cases with unknown recovery status one year after diagnosis of heart failure, (II) cases treated with trastuzumab, and (III)** **patients with a second malignancy**

|  | **Median value (IQR)** | **Cases** | | **Controls** | | | **RR** | **95% CI** | | ***p*** |  |
| --- | --- | --- | --- | --- | --- | --- | --- | --- | --- | --- | --- |
| **Additional model I: excluding cases with unknown recovery status one year after HF diagnosis^*^** |  |  |  |  | |  |  |  | |  |  |
| **Total** |  | 96 (N) | 100 (%) | 288 (N) | 100 (%) | |  |  |  | | |
| **Radiotherapy** |  |  |  |  |  | |  |  |  | | |
| Mean heart dose 0-1 Gy | 0.4 Gy (0.2-0.9) | 14 | 14.6 | 36 | 12.5 | | 1.0^†^ | 0.37-1.8 |  | | |
| Mean heart dose 2-9 Gy | 4.3 Gy (3.8-6.7) | 23 | 24.0 | 82 | 28.5 | | 0.8 | 0.49-2.6 | *0.61* | | |
| Mean heart dose ≥10 Gy | 14.7 Gy (13.8-17.1) | 27 | 28.1 | 81 | 28.1 | | 1.1 | 0.55-3.4 | *0.79* | | |
| No radiotherapy |  | 31 | 32.3 | 83 | 28.8 | | 1.4 | 0.03-2.6 | *0.50* | | |
| Mean heart dose unknown^‡^ |  | 1 | 1.0 | 6 | 2.1 | | - | - | | *-* |  |
| **Chemotherapy** |  |  |  |  |  | |  |  | |  |  |
| No chemotherapy |  | 43 | 44.8 | 176 | 61.1 | | 1.0^†^ | 0.61-1.6 | |  |  |
| CMF-like |  | 9 | 9.4 | 48 | 16.7 | | 0.7 | 0.31-1.5 | | *0.38* |  |
| Anthracyclines |  | 34 | 35.4 | 52 | 18.1 | | 6.7 | 3.3-13.6 | | *<0.001* |  |
| Anthracyclines & trastuzumab |  | 8 | 8.3 | 7 | 2.4 | | 17.5 | 5.0-61.3 | | *<0.001* |  |
| Other type of chemotherapy or type unknown |  | 2 | 2.1 | 5 | 1.7 | | 3.0 | 0.48-18.8 | | *-* |  |
| **Endocrine** **therapy** |  |  |  |  |  | |  |  | |  |  |
| No endocrine therapy |  | 62 | 64.6 | 229 | 79.5 | | 1.0^†^ | 0.66-1.5 | |  |  |
| Tamoxifen |  | 20 | 20.8 | 37 | 12.9 | | 1.6 | 0.82-3.0 | | *0.24* |  |
| Tamoxifen & aromatase inhibitors |  | 7 | 7.3 | 15 | 5.2 | | 1.4 | 0.47-3.9 | | *0.61* |  |
| Aromatase inhibitors |  | 6 | 6.3 | 5 | 1.7 | | 3.5 | 0.86-14.2 | | *0.10* |  |
| Type of endocrine therapy unknown^§^ |  | 1 | 1.0 | 2 | 0.7 | | - | - | | *-* |  |
| **Additional model II: excluding patients treated with trastuzumab** |  |  |  |  |  | |  |  | |  |  |
| **Total** |  | 88 (N) | 100 (%) | 259 (N) | 100 (%) | |  |  | |  |  |
| **Radiotherapy** |  |  |  |  |  | |  |  | |  |  |
| Mean heart dose 0-1 Gy | 0.4 Gy (0.2-0.9) | 13 | 14.8 | 33 | 12.7 | | 1.0^†^ | 0.50-2.0 | |  |  |
| Mean heart dose 2-9 Gy | 3.9 Gy (3.8-6.9) | 18 | 20.5 | 62 | 23.9 | | 0.8 | 0.41-1.6 | | *0.70* |  |
| Mean heart dose ≥10 Gy | 14.7 Gy (14.2-17.1) | 25 | 28.4 | 76 | 29.3 | | 0.6 | 0.36-0.99 | | *0.25* |  |
| No radiotherapy |  | 31 | 35.2 | 82 | 31.7 | | 0.9 | 0.54-1.4 | | *0.75* |  |
| Mean heart dose unknown^‡^ |  | 1 | 1.1 | 6 | 2.3 | | - | - | | *-* |  |
| **Chemotherapy** |  |  |  |  |  | |  |  | |  |  |
| No chemotherapy |  | 43 | 48.9 | 170 | 65.6 | | 1.0^†^ | 0.69-1.4 | |  |  |
| CMF-like |  | 9 | 10.2 | 48 | 18.5 | | 0.7 | 0.32-1.5 | | *0.40* |  |
| Anthracyclines |  | 34 | 38.6 | 39 | 15.1 | | 7.7 | 3.4-17.5 | | *<0.001* |  |
| Other type of chemotherapy or type unknown |  | 2 | 2.3 | 2 | 0.8 | | - |  | |  |  |
| **Endocrine** **therapy**^§^ |  |  |  |  |  | |  |  | |  |  |
| No endocrine therapy |  | 58 | 65.9 | 211 | 81.5 | | 1.0^†^ | 0.63-1.6 | |  |  |
| Tamoxifen |  | 20 | 22.7 | 34 | 13.1 | | 1.5 | 0.79-2.8 | | *0.33* |  |
| Tamoxifen & aromatase inhibitors |  | 5 | 5.7 | 9 | 3.5 | | 1.0 | 0.30-3.9 | | *0.95* |  |
| Aromatase inhibitors |  | 4 | 4.6 | 3 | 1.2 | | 4.6 | 0.79-27.2 | | *0.10* |  |
| Type of endocrine therapy unknown^§^ |  | 1 | 1.1 | 2 | 0.8 | | - | - | | *-* |  |
|  |  |  |  |  |  | |  |  | |  |  |
|  |  |  |  |  |  | |  |  | |  |  |
|  |  |  |  |  |  | |  |  | |  |  |
|  |  |  |  |  |  | |  |  | |  |  |
| **Supplemental Table 6. Continued** |  |  |  |  |  | |  |  | |  |  |
|  | **Median value (IQR)** | **Cases** | | **Controls** | | | **RR** | **95% CI** | | ***p*** |  |
| **Additional model III: excluding patients with a second malignancy prior to cut-off date/ diagnosis of heart failure** |  |  |  |  |  | |  |  | |  |  |
| **Radiotherapy** |  |  |  |  |  | |  |  | |  |  |
| Median mean heart dose^\|\|^ (IQR) |  | 6.8 Gy (0.9-12.9) | | 3.8 Gy (0.2-12.9) | | |  |  | |  |  |
| Mean heart dose 0-1 Gy | 0.4 Gy (0.2-0.9) | 25 | 30.5 | 93 | 31.5 | | 1.0^†^ | 0.52-1.9 | |  |  |
| Mean heart dose 2-9 Gy | 4.3 Gy (3.8-6.8) | 22 | 26.8 | 80 | 27.1 | | 0.7 | 0.43-1.2 | | *0.42* |  |
| Mean heart dose ≥10 Gy | 14.6 Gy (13.4-16.8) | 24 | 29.3 | 78 | 26.4 | | 1.0 | 0.59-1.7 | | *0.97* |  |
| No radiotherapy |  | 10 | 12.2 | 38 | 12.9 | | 1.3 | 0.63-3.0 | | *0.64* |  |
| Mean heart dose unknown^‡^ |  | 1 | 1.2 | 6 | 2.0 | | - | - | | - |  |
| **Chemotherapy** |  |  |  |  |  | |  |  | |  |  |
| No chemotherapy |  | 31 | 37.8 | 179 | 60.7 | | 1.0^†^ | 0.55-1.8 | |  |  |
| CMF-like |  | 8 | 9.8 | 44 | 14.9 | | 0.9 | 0.38-2.1 | | *0.81* |  |
| Anthracyclines^§^ |  | 30 | 36.6 | 59 | 20.0 | | 7.0 | 3.5-13.9 | | *<0.001* |  |
| Anthracyclines & trastuzumab^§^ |  | 11 | 13.4 | 7 | 2.4 | | 26.5 | 8.0-88.3 | | *<0.001* |  |
| Other type of chemotherapy or type unknown |  | 2 | 2.4 | 6 | 2.0 | | 5.9 | 0.89-38.5 | | *0.08* |  |
| **Endocrine** **therapy** |  |  |  |  |  | |  |  | |  |  |
| No endocrine therapy |  | 49 | 59.8 | 236 | 80.0 | | 1.0^†^ | 0.64-1.6 | |  |  |
| Tamoxifen |  | 17 | 20.7 | 36 | 12.1 | | 1.5 | 0.77-3.0 | | *0.31* |  |
| Tamoxifen & aromatase inhibitors |  | 7 | 8.5 | 17 | 5.9 | | 1.7 | 0.58-4.9 | | *0.37* |  |
| Aromatase inhibitors |  | 8 | 9.8 | 4 | 1.4 | | 5.4 | 1.3-22.2 | | *0.03* |  |
| Type of endocrine therapy unknown^§^ |  | 1 | 1.2 | 2 | 0.7 | | - | - | | *-* |  |

Abbreviations: IQR, interquartile range; RR, rate ratio; CI, confidence interval; Gy, Gray.
^*^ Analysis excluding six heart failure cases treated with trastuzumab for whom recovery of ejection fraction was unknown in the first year after heart failure diagnosis.
^†^ Reference category
^‡^ Heart doses were unknown for 7 patients (1 case, 6 controls) because their radiotherapy charts were unavailable
^§^Type of endocrine therapy was unknown for 3 patients (1 case, 2 controls).

^||^ In patients treated with radiotherapy.

^¶^ Anthracycline treatment consisted of an epirubicin-containing regimen for 12/41 cases and 15/66 controls, and of a doxorubicin-containing regimen for 28/41 cases and 52/66 controls.

**Supplemental table 7. Time between breast cancer diagnosis and heart failure diagnosis by type of chemotherapy and trastuzumab treatment**

|  | | **No chemotherapy** | | **CMF-like regimens** | | **Anthacyclines, no trastuzumab** | | **Anthracyclines, trastuzumab** | | **Type unknown** | | **Total** |
| --- | --- | --- | --- | --- | --- | --- | --- | --- | --- | --- | --- | --- |
| **Years since breast cancer diagnosis** |  | |  | |  | |  | |  | |  | |
| <1 year | | 0 | | 0 | | 1 | | 0 | | 0 | | 1 |
| 1 year | | 0 | | 0 | | 1 | | 12 | | 0 | | 13 |
| 2 years | | 0 | | 0 | | 6 | | 1 | | 0 | | 7 |
| 3 years | | 1 | | 0 | | 2 | | 1 | | 0 | | 4 |
| 4 years | | 0 | | 0 | | 2 | | 0 | | 1 | | 3 |
| 5-9 years | | 4 | | 0 | | 10 | | 0 | | 1 | | 15 |
| 10-19 years | | 21 | | 5 | | 11 | | 0 | | 0 | | 37 |
| 20-30 years | | 18 | | 4 | | 0 | | 0 | | 0 | | 22 |
|  | |  | |  | |  | |  | |  | |  |
| **Total** | | 44 | | 9 | | 33 | | 14 | | 2 | | 102 |

| **Supplemental table 8. Associations between estimated mean left ventricular dose and heart failure risk** | | | | | | | |  |  |  |
| --- | --- | --- | --- | --- | --- | --- | --- | --- | --- | --- |
|  | **Median value (IQR)** | **Cases** | | **Controls** | | **RR** | **95% CI** | | ***p*** | |
| **Total** |  | 102 (N) | 100 (%) | 306 (N) | 100 (%) |  |  | |  | |
| **Estimated mean left ventricular dose** |  |  |  |  |  |  |  | |  | |
| **Patients not treated with anthracyclines or trastuzumab** |  |  |  |  |  |  |  | |  | |
| Median mean left ventricular dose (IQR) |  | 1.0 Gy (0.3-5.0) | | 1.3 Gy (0.5-5.8) | |  |  | |  | |
| Total |  |  |  |  |  |  |  | |  | |
| No radiotherapy |  | 11 | 20.4 | 33 | 14.0 | 1.0^†^ | 0.48-2.1 | |  | |
| 0-4 Gy | 1.0 Gy (0.1-1.3) | 29 | 53.7 | 125 | 53.0 | 0.8 | 0.52-1.2 | | *0.57* | |
| ≥5 Gy | 6.4 Gy (5.8-12.3) | 13 | 24.1 | 76 | 32.2 | 0.5 | 0.28-0.93 | | *0.16* | |
| Mean heart dose unknown^‡^ |  | 1 | 1.9 | 2 | 0.9 | - | - | |  | |
| **Patients treated with anthracyclines but not trastuzumab** |  |  |  |  |  |  |  | |  | |
| Median mean left ventricular dose (IQR) |  | 0.9 Gy (0.4-7.5) | | 1.3 Gy (0.1-6.0) | |  |  | |  | |
| Total |  |  |  |  |  |  |  | |  | |
| No radiotherapy |  | 2 | 5.9 | 5 | 8.3 | 1.0^†^ | 0.18-5.4 | |  | |
| 0-4 Gy | 0.6 Gy (0.0-1.3) | 21 | 61.8 | 36 | 60.0 | 1.0 | 0.63-1.7 | | *0.98* | |
| ≥5 Gy | 7.5 Gy (7.4-0.9) | 11 | 32.4 | 15 | 25.0 | 0.8 | 0.35-2.0 | | *0.85* | |
| Mean heart dose unknown^‡^ |  | 0 | 0.0 | 4 | 6.7 | - | - | |  | |
| **Patients treated with trastuzumab** |  |  |  |  |  |  |  | |  | |
| Median mean heart dose^*,†^ (IQR) |  | 0.3 Gy (0.0-1.3) | | 0.8 Gy (0.3-7.5) | |  |  | |  | |
| Total |  | 14 |  | 10 |  |  |  | |  | |
| Abbreviations: RR, rate ratio; CI, confidence interval; Gy, Gray.  Model additionally included categorical variables for type of chemotherapy (no chemotherapy, CMF-like regimen, anthracyclines, anthracyclines & trastuzumab) and endocrine therapy (no, yes).  ^†^ Reference category  ^‡^  Heart doses were unknown for 7 patients (1 case, 6 controls) because their radiotherapy charts were unavailable | | | | | | | | | |  |

| **Supplemental table 9. Associations between patient-related risk factors at breast cancer diagnosis and heart failure risk** | | | | | | |  |
| --- | --- | --- | --- | --- | --- | --- | --- |
|  | **Cases** |  | **Controls** |  |  |  |  |
| **Total** | 102 (N) | 100 (%) | 306 | 100 (%) | **RR** | **95% CI** | ***p*** |
| **Risk factor** |  |  |  |  |  |  |  |
| **Smoking** |  |  |  |  |  |  |  |
| Not smoking at breast cancer diagnosis | 65 | 63.7 | 179 | 58.5 | 1.0^†^ | 0.77-1.3 |  |
| Smoking at breast cancer diagnosis | 31 | 30.4 | 90 | 29.4 | 0.9 | 0.55-1.4 | *0.57* |
| Unknown | 6 | 5.9 | 37 | 12.1 | 0.4 | 0.16-1.0 | *0.07* |
| **Body mass index** |  |  |  |  |  |  |  |
| Median (IQR) | 24.3 | 22.0-27.4 | 23.7 | 22.0-26.0 |  |  |  |
| Normal weight (BMI<25) | 41 | 40.2 | 139 | 45.4 | 1.0^†^ | 0.71-1.4 |  |
| Overweight (BMI 25-29) | 24 | 23.5 | 63 | 20.6 | 1.3 | 0.77-2.1 | *0.45* |
| Obese (BMI ≥30) | 11 | 10.8 | 16 | 5.2 | 2.3 | 1.0-5.4 | *0.07* |
| Unknown | 26 | 25.5 | 88 | 28.8 | 1.1 | 0.70-1.7 | *0.80* |
| **Comorbidity** |  |  |  |  |  |  |  |
| No diabetes | 101 | 99.0 | 302 | 98.7 | 1.0^†^ |  |  |
| Diabetes | 1 | 1.0 | 4 | 1.3 | 0.4 | 0.03-5.6 | *0.53* |
| No hypertension | 98 | 96.1 | 290 | 94.8 | 1.0^†^ |  |  |
| Hypertension | 4 | 3.9 | 16 | 5.2 | 0.6 | 0.19-1.9 | *0.37* |
| No AP, COPD and CVA | 100 | 98.0 | 304 | 99.4 | 1.0^†^ |  |  |
| AP, COPD or CVA | 2 | 2.0 | 2 | 0.7 | 3.5 | 0.40-31.1 | *0.26* |
| **Menopausal** **status** |  |  |  |  |  |  |  |
| Premenopausal | 42 | 41.2 | 145 | 47.4 | 1.0^†^ | 0.58-1.7 |  |
| Perimenopausal | 11 | 10.8 | 21 | 6.9 | 2.2 | 1.0-4.8 | *0.10* |
| Postmenopausal | 36 | 35.3 | 107 | 35.0 | 1.3 | 0.77-2.1 | *0.55* |
| Unknown | 13 | 12.8 | 33 | 10.8 | 1.9 | 0.98-3.6 | *0.14* |

Abbreviations: RR, rate ratio; CI, confidence interval; IQR, interquartile range; BMI, body mass index; AP, angina pectoris; COPD, chronic obstructive pulmonary disease; CVA, cerebrovascular accident.

Model included the following variables: smoking (never smoked; smoking at breast cancer diagnosis; previous smoker; does not smoke, previous smoking not stated; unknown), body mass index (normal weight [BMI<25], overweight [BMI 25-29], obese [BMI≥30], unknown); diabetes (no, yes); hypertension (no, yes); angina pectoris, chronic obstructive pulmonary disease or cerebrovascular accident (no, yes); menopausal status (premenopausal, perimenopausal, postmenopausal, unknown).

^†^ Reference category
